# Characteristics of patients with venous thromboembolism and atrial fibrillation in Venezuela

**DOI:** 10.1186/1471-2458-11-415

**Published:** 2011-05-31

**Authors:** Dimitri Bennett, Jorge Abate, Page E Abrahamson

**Affiliations:** 1 WWEpidemiology, GlaxoSmithKline, 1250 South Collegeville road, Collegeville. PA 19426, USA; 2 Data Management & Biostatistics, Research & Data Processing, C.A., Torre "Anexo D", Piso 6, Oficina PH-C, Av. Sorocaima, San Bernardino, Caracas, 1010, Venezuela

## Abstract

**Background:**

Studies describing venous thromboembolic event (VTEE) and atrial fibrillation (AF) in South American populations are limited. The aim of this cross-sectional study was to describe the characteristics of Venezuelan patients admitted and treated for these conditions.

**Methods:**

A retrospective medical record review of 1397 consecutive patients admitted to three private hospitals or clinics between January 2000 and December 2005 was performed. Data was collected on demographics, anthropometrics, hospital visit, comorbidities and treatment.

**Results:**

Among 401 VTEE and 996 AF patients, men were more likely to have AF (58%) while more women experienced a VTEE (58%). Most patients were admitted via the emergency room (87%) and had only one event during the study period (83%). Common comorbidities included hypertension (46%), heart failure (17%), diabetes (12%) and congestive heart failure (11%). Characteristics of Venezuelan patients with VTEE and AF are similar to that reported in the literature for other populations.

**Conclusions:**

These results provide background characteristics for future studies assessing risk factors for AF and VTEE in South American populations.

## Background

The size of the elderly population is growing rapidly worldwide, including developed and developing countries [1]. Older age is a risk factor for developing many diseases, including venous thromboembolic events (VTEE) and atrial fibrillation (AF). Thus, the disease burden associated with VTEE and AF is expected to expand as elderly populations continue to rise.

The impact of VTEE and AF on mortality and morbidity is substantial, as are the socioeconomic consequences in terms of hospital admissions and management of chronic diseases and disabilities. AF is the most common cardiac rhythm disorder, affecting approximately 2.3 million people in the United States (US) and 4.8 million in the 6 major pharmaceutical markets (France, Spain, Germany, United Kingdom, Italy, Japan,) combined [2]. VTEEs are common vascular diseases with an average annual incidence rate among in the US and Europe of 100 to 200 per 100,000 person-years in the US and Europe; roughly 900,000 incident or recurrent cases occurring annually in the US alone [3-6]. The incidence rate appears to be similar or higher among African-Americans and lower for Asian- and Native-American populations in the US [7,8].

Although the epidemiology of VTEE and AF is very well described in North America and Europe, there is a paucity of published epidemiological data on the descriptive epidemiology of patients with these conditions in South American populations. Therefore, we conducted a descriptive study of patients diagnosed with VTEE and AF to better understand the disease burden in Venezuela.

## Methods

Using a retrospective cross-sectional study design, we reviewed medical records for two groups of adult (18+ years) patients (VTEE or AF) diagnosed between January 2000 and December 2005 at three private Venezuelan hospitals or clinics located in Caracas: Clínica Santa Sofía, Policlínica Metropolitana, and Hospital de Clínicas Caracas. Hospital de Clinicas Caracas, the most technologically advanced private health care institution in Venezuela with 13,500 admissions, 7,000 annual surgeries and 180 beds for inpatient population. The hospital provides primary, tertiary, and transplant services and employs 400 physicians and 373 nurses who provide excellent medical care services. Policlinica Metropolitana and Clinica Santa Sofia are another 2 private clinics that provide excellent medical services. Both clinics provide primary, tertiary and transplant services as well. All hospitals and clinics included in the study maintain a central repository of electronic medical records for all patients admitted through the emergency rooms. The diagnosis of VTEE or AF was defined using the International Classification of Disease 10^th ^Revision (ICD- 10) and confirmed by the treating physician [9]. This research received a waiver of consent from the Independent Ethics Committee ("Comisión de Bioética y Bioseguridad del Fondo Nacional de Ciencia y Tecnología") in Venezuela for use of patient medical record data.

Data was abstracted from medical records using a structured case report form to gather information regarding age, gender, weight, source at index hospitalization, admission diagnosis, use of anticoagulation therapy, length of hospital stay and disposition, comorbidities (defined by ICD-10 codes) and medications (Venezuela's Vademecum). Vademecum is a medication handbook which contains information on trademarks, active principles, interactions, adverse reactions, international equivalences, and manufacturers. Descriptive statistics were calculated using SPSS V. 12 software.

## Results

The study population included 401 patients with VTEEs and 996 AF patients (Table 1). The most common VTEEs were phlebitis or thrombophlebitis (67%), pulmonary embolism (17%), other venous embolism or thrombosis (9%), venous embolism combined with phlebitis (6%). The mean age at diagnosis was 60.1 and 70.7 years old for VTEE and AF patients, respectively. More VTEEs occurred among women (58%), whereas AF was more common in men (58%). Most patients presented at the emergency room (VTEE: 89%, AF: 87%) with mean hospitalization duration of approximately 5 days for both patient groups. The majority of patients experienced a single VTEE (88%) or AF (80%) event during the study period from 2000 to 2005. Most patients returned home following hospitalization; 5 VTEE and 4 AF patients readmitted to the hospital (data not shown). Comorbidities were generally more common among AF patients compared to patients diagnosed with a VTEE. Hypertension was the most common comorbidity observed in both VTEE (26%) and AF (54%) patients, followed by heart failure (VTEE: 3%, AF: 23%), diabetes (VTEE: 12%, AF: 12%) and congestive heart failure (VTEE: 3%, AF: 14%). Cancer more common among VTEE patients (8%) compared with AF patients (4%). In a subset of patients with anthropometric data available, mean height and weight were similar in both disease groups (data not shown).

**Table 1:** Characteristics of patients diagnosed with a venous thromboembolic event (VTEE) or atrial fibrillation (AF), Venezuela, 2000-2005

Characteristic	VTEE (n = 401)	AF (n = 996)
**Age (years)**, mean (range)	60.1 (22-103)	70.7 (18-101)
**Sex, N (%)**		
Male	168 (42%)	577 (58%)
Female	230 (58%)	410 (42%)
**No. VTEE/AF Events, 2000- 2005, N (%)**		
1	354 (88%)	798 (80%)
2	37 (9%)	146 (15%)
3	8 (2%)	35 (3%)
4+	2 (<1%)	17 (2%)
**Hospital Admission Source, N (%)**		
Emergency room	356 (89%)	865 (87%)
Routine admission/outpatient	27 (7%)	76 (8%)
Transfer	16 (4%)	51 (5%)
Unknown	2 (<1%)	4 (<1%)
**Days of Hospital Stay, mean (range)**	5.4 (1-75)	4.5 (1-49)
**Comorbidities, N (%)**		
Hypertension	105 (26%)	540 (54%)
Heart failure	13 (3%)	225 (23%)
Diabetes	46 (12%)	122 (12%)
Congestive heart failure	12 (3%)	134 (14%)
Cancer	33 (8%)	35 (4%)
Coronary artery disease	4 (1%)	47 (5%)
Prior myocardial infarction	2 (<1%)	45 (5%)
Prior transient ischemic attack	2 (<1%)	37 (4%)
Prior stroke	4 (1%)	29 (3%)
Prior bleeding event	0	2 (<1%)

Table 2 describes the primary hospital admission and discharge codes for the VTEE and AF patients. Deep vein thromboembolism was the most common hospital admission (41%) and discharge (53%) diagnosis for VTEE patients. Among AF patients, AF was listed as the admission diagnosis for only 44% of population; however, AF was recorded as the primary diagnosis for 86% of patients at the time of hospital discharge.

**Table 2:** Common hospital admission and discharge diagnoses for patients with venous thromboembolic event (VTEE) and atrial fibrillation (AF) in Venezuela

	VTEE (n = 401)		AF (n = 996)	
	
	n	%	n	%
**Admission diagnosis**				
Atrial fibrillation	0	0.0%	439	44.1%
Deep venous thromboembolism	165	41.1%	0	0.0%
Cerebrovascular accident	6	1.5%	86	8.6%
Cardiac insufficiency	8	2.0%	60	6.0%
Pulmonary embolism	45	11.2%	0	0.0%
Thrombophlebitis	34	8.5%	0	0.0%
Ischemic cardiopathy	3	0.7%	27	2.7%
Pneumonia	6	1.5%	21	2.1%
Unstable angina	0	0.0%	27	2.7%
Respiratory infection	4	1.0%	23	2.3%
Hypertension	5	1.2%	22	2.2%
Respiratory insufficiency	12	3.0%	14	1.4%
Cellulitis	25	6.2%	0	0.0%
Embolism/thromb. of arteries lower extrem.	21	5.2%	0	0.0%
Arrhythmia	0	0.0%	14	1.4%
Syncope	0	0.0%	14	1.4%
Pulmonary oedema	0	0.0%	12	1.2%
Acute coronary syndrome	1	0.2%	11	1.1%
Other	66	16.5%	226	22.7%
**Discharge diagnosis**				
Atrial fibrillation	0	0.0%	857	86.0%
Deep venous thromboembolism	212	52.9%	3	0.3%
Pulmonary embolism	64	16.0%	7	0.7%
Thrombophlebitis	47	11.7%	0	0.0%
Cardiac insufficiency	6	1.5%	38	3.8%
Cerebrovascular accident	4	1.0%	24	2.4%
Venous embolism and thrombosis	23	5.7%	1	0.1%
Ischemic cardiopathy	5	1.2%	14	1.4%
Auricular flutter	0	0.0%	14	1.4%
Other	40	10.0%	39	3.9%

As presented in Table 3, enoxaparin and warfarin were the mostly commonly prescribed anticoagulant medications for both VTEE and AF, although a larger proportion of VTEE patients (66% and 68%, respectively) were prescribed these medications compared to AF patients (48% and 32%). Heparin was used more frequently among VTEE patients (40%) compared to those with AF (3%); roughly 3% of both patient groups were treated with dalteparin. The most common concomitant medications among the VTEE patients were proton-pump inhibitors (38%), H2 blockers (33%), and benzodiazepines (30%). A similar proportion of AF patients were treated with these same medications, but the majority was also treated with antiarrhythmic drugs (83%). Twenty (2%) of AF patients and 3 (<1%) of VTEE patients died during the study period (data not shown).

**Table 3:** Common medications prescribed for patients with a venous thromboembolic event (VTEE) or atrial fibrillation (AF)

	VTEE (n = 401)		AF (n = 996)	
	
	n	%	n	%
**Anticoagulant used**				
Dalteparin sodium	12	3.0%	24	2.4%
Enoxaparin	264	65.8%	476	47.8%
Heparin sodium	159	39.7%	32	3.2%
Warfarin sodium	271	67.6%	323	32.4%
**Class of Concomitant Medications**				
Antiarrhythmic drugs	38	9.5%	831	83.4%
Proton-pump inhibitors	151	37.7%	293	29.4%
Benzodiazepines	119	29.7%	312	31.3%
H2 blockers	131	32.7%	239	24.0%
Diuretics	22	5.5%	248	24.9%
Antiplatelet drugs	51	12.7%	188	18.9%
Angiotensin-converting enzyme inhibitor	27	6.7%	208	20.9%
Beta blockers	25	6.2%	156	15.7%
Aldosterone receptor antagonists	15	3.7%	141	14.2%
Nonsteroidal anti-inflammatory medications	66	16.5%	73	7.3%
Salicylates	17	4.2%	85	8.5%
Quinolone antibiotics	31	7.7%	54	5.4%
Calcium antagonist	9	2.2%	71	7.1%
Cephalosporins	44	11.0%	26	2.6%

## Discussion

This is one of the first studies describing the epidemiology of VTEE and AF in Venezuela. The frequency of some common and rare comorbid conditions in the Venezuelan patient population is similar to what has been observed in North American and European populations. For example, approximately one quarter of VTEE and half of all AF patients in the Venezuelan population were hypertensive; similar rates have been reported in the literature [10-14]. Rare events such as prior myocardial infarction, transient ischemic attack or stroke each occurred in roughly 0-5% of AF patients in the present study as well as other large cohorts [11,12,14]. The prevalence of heart failure and diabetes found in Venezuelan VTEE and AF populations also is similar to what has been reported in other published studies [10-15]. This new research suggests that Venezuelan patients with VTEE and AF share similar epidemiologic characteristics and risk factors for developing these conditions as populations in other parts of the world.

We found that the Venezuelan VTEE population was less likely to have cancer as a comorbidity (8%) compared to VTEE patients in the US and Europe which ranges from 20-32% [10,15,16]. It is estimated that cancer accounts for approximately 20% of incident VTEE in the US and the risk is highest for patients diagnosed with certain types of cancer or receiving particular therapies such as immunosuppressive or cytotoxic chemotherapy [3]. It is possible that the lower cancer prevalence in Venezuelans with VTEE is explained by reduced access to expensive or experimental cancer treatments or differences in incidence rates of cancers such as digestive malignancies that are more closely associated with VTEE [3,17].

There are several strengths to this cross-sectional study. First, the retrospective study included the use of ICD-10 diagnosis codes and Vademecum medication codes which provide consistent patient data. The use of ICD codes has been shown to reliably capture cardiovascular and cerebrovascular events [18,19]. For example, defining patient-level covariates by the ICD-10 diagnoses are quite good and coded with high sensitivity for coronary artery disease/ischemic heart disease (98%), diabetes mellitus (99%), history of cerebrovascular accident (95%), hypertension (83%), and TIA (79%) [20,21]. Further, we studied data from the major private hospitals or clinics serving the Caracas population.

This study has several limitations. As with most studies based on medical record data, this study was limited by the information recorded in the physicians' notes. We did not validate the data about the sensitivity or specificity of the ICD-10 codes used to identify VTEE or AF patients. Additionally, no other standard clinical criteria or definitions beyond ICD-10 codes were used to define the VTEE or AF events. Thus, the completeness of the captured VTEE or AF events is unclear. However, the ICD-10 classification system includes distinct codes for the diseases of interest, reducing the likelihood of misclassification. Previous studies have reported high sensitivity associated with ICD-10 discharge codes for PE (89%) and AF (98%); the sensitivity for DVT diagnoses is lower (58%) [22,20]. It is unclear whether the patients from the three hospitals and clinics are representative of the overall population in Venezuela. For example, these clinics may represent a higher socioeconomic bracket than the average for Venezuela. This study does not include an internal comparison group to determine whether the characteristics of VTEE or AF patients significantly differ from the general population.

Little is known about VTEE or AF in regions outside the United States or Europe. This study provides a starting point for additional future analyses of these diseases in South American populations. Accurately assessing the region-specific disease burden, patient characteristics and treatment patterns is essential for understanding the unmet needs and improving public health.

## Conclusions

In conclusion, the study provides evidence that Venezuelan patients diagnosed with VTEE and AF events are similar with regard to epidemiological and clinical factors as North American and European patients.

## Competing interests

DB is an employee with GlaxoSmithKline.

## Authors' contributions

All listed authors meet the criteria for authorship set forth by the International Committee for Medical Journal Editors. DB and JA led all aspects of the study and drafted the manuscript. DB, JA and PEA analyzed and interpreted the data and revised the manuscript. All authors read and approved the final manuscript.

## Pre-publication history

The pre-publication history for this paper can be accessed here:

http://www.biomedcentral.com/1471-2458/11/415/prepub

## References

[B1] Lutz W Sanderson W Scherbov S The coming acceleration of global population ageing Nature 2008 451 716 719 10.1038/nature06516 18204438

[B2] Silverstein MD Heit JA Mohr DN Petterson TM O'Fallon WM Melton LJ III Trends in the incidence of deep vein thrombosis and pulmonary embolism: a 25-year population-based study Arch Intern Med 1998 158 585 93 10.1001/archinte.158.6.585 9521222

[B3] Heit JA Venous thromboembolism: disease burden, outcomes and risk factors J Thromb Haemost 2005 3 1611 1617 10.1111/j.1538-7836.2005.01415.x 16102026

[B4] Heit JA The Epidemiology of Venous Thromboembolism in the Community Arterioscler Thromb Vasc Biol 2008 28 370 2 10.1161/ATVBAHA.108.162545 18296591 PMC2873781

[B5] Næss IA Christiansen SC Romundstad P Cannegieter SC Rosendaal FR Hammerstrøm J Incidence and mortality of venous thrombosis: a population-based study J Thromb Haemost 2007 5 692 9 10.1111/j.1538-7836.2007.02450.x 17367492

[B6] Oger E Incidence of venous thromboembolism: a community-based study in Western France. EPI-GETBP Study Group. Groupe d'Etude de la Thrombose de Bretagne Occidentale Thromb Haemost 2000 83 657 60 10823257

[B7] Hooper WC Holman RC Heit JA Cobb N Venous thromboembolism hospitalizations among American Indians and Alaska Natives Thromb Res 2002 108 273 8 10.1016/S0049-3848(03)00058-6 12676185

[B8] White RH Zhou H Romano PS Incidence of idiopathic deep venous thrombosis and secondary thromboembolism among ethnic groups in California Ann Intern Med 1998 128 737 40 9556467 10.7326/0003-4819-128-9-199805010-00006

[B9] International Classification of Diseases 10th Revision (ICD-10) 2006 World Health Organization

[B10] Dowling NF Austin H Dilley A Whitsett C Evatt BL Hooper WC The epidemiology of venous thromboembolism in Caucasians and African-Americans: the GATE Study J Thromb Haemost 2002 1 80 87 10.1046/j.1538-7836.2003.00031.x 12871543

[B11] Harley CR Riedel AA Hauch O Nelson M Wygant G Reynolds M Anticoagulation therapy in patients with chronic atrial fibrillation: a retrospective claims data analysis Curr Med Res Opin 2005 21 215 222 10.1185/030079904X20321 15801992

[B12] Dagres N Nieuwlaat R Vardas PE Andresen D Lévy S Cobbe S Kremastinos DTh Breithardt G Cokkinos DV Crijns HJGM Gender-Related Differences in Presentation, Treatment, and Outcome of Patients With Atrial Fibrillation in Europe A Report From the Euro Heart Survey on Atrial Fibrillation J Am Coll Cardiol 2007 49 572 7 10.1016/j.jacc.2006.10.047 17276181

[B13] Walker AM Bennett D Epidemiology and outcomes in patients with atrial fibrillation in the United States Heart Rhythm 2008 5 1365 1372 10.1016/j.hrthm.2008.07.014 18929320

[B14] Parkash R Green MS Kerr CR Connolly SJ Klein GJ Sheldon R Talajic M Dorian P Humphries KH The association of left atrial size and occurrence of atrial fibrillation: A prospective cohort study from the Canadian Registry of Atrial Fibrillation Am Heart J 2004 148 649 54 10.1016/j.ahj.2004.04.029 15459596

[B15] Spencer FA Emery C Joffe SW Pacifico L Lessard D Reed G Gore JM Goldberg RJ Incidence rates, clinical profile, and outcomes of patients with venous thromboembolism. The Worcester VTE study J Thromb Thrombolysis 2009 28 401 9 10.1007/s11239-009-0378-3 19629642 PMC3248815

[B16] Arcelus JI Caprini JA Monreal M Suárez C González-Fajardo J The management and outcome of acute venous thromboembolism: A prospective registry including 4011 patients J Vasc Surg 2003 38 916 22 10.1016/S0741-5214(03)00789-4 14603194

[B17] Bosetti C Malvezzi M Chatenoud L Negri E Levi F La Vecchia C Trends in cancer mortality in the Americas, 1970-2000 Ann Oncol 2005 16 489 511 10.1093/annonc/mdi086 15668262

[B18] Kirkman MA Manhattanakul W Gregson BA Mendelow AD The accuracy of hospital discharge coding for hemorrhagic stroke Acta Neurol Belg 2009 109 114 9 19681442

[B19] Birman-Deych E Waterman AD Yan Y Nilasena DS Radford MJ Gage BF Accuracy of ICD-9-CM codes for identifying cardiovascular and stroke risk factors Med Care 2005 43 480 5 10.1097/01.mlr.0000160417.39497.a9 15838413

[B20] Casez P Labarere J Sevestre MA Haddouche M Courtois X Mercier S Lewandowski E Fauconnier J François P Bosson JL ICD-10 hospital discharge diagnosis codes were sensitive for identifying pulmonary embolism but not deep vein thrombosis J Clin Epidemiol 2010 63 790 7 10.1016/j.jclinepi.2009.09.002 19959332

[B21] Ghia D Thomas PR Cordato D Worthington JM Cappelen-Smith C Griffith N Hanna I Hodgkinson SJ McDougall A Beran RG Validation of Emergency and Final Diagnosis Coding in Transient Ischemic Attack: South Western Sydney Transient Ischemic Attack Study Neuroepidemiology 2010 35 53 58 10.1159/000310338 20431303

[B22] Kokotailo RA Hill MD Coding of stroke and stroke risk factors using international classification of diseases, revisions 9 and 10 Stroke 2005 36 1776 81 10.1161/01.STR.0000174293.17959.a1 16020772

